# Primary squamous cell carcinoma of the urinary bladder presenting as an inguinal mass

**DOI:** 10.4102/sajr.v25i1.2048

**Published:** 2021-03-29

**Authors:** Zahra Qaiyumi, Pankaj Nepal, Christopher Iannuzzi, Joshua Sapire

**Affiliations:** 1 Frank H. Netter MD School of Medicine, Quinnipiac University, North Haven, CT, The United States of America; 2 Department of Radiology, St. Vincent’s Medical Center, Bridgeport, CT, The United States of America; 3 Department of Radiation Oncology, St. Vincent’s Medical Center, Bridgeport, CT, The United States of America

**Keywords:** urinary bladder hernia, squamous cell carcinoma, imaging, CT, PET-CT

## Abstract

This report involves a rare case of a 74-year-old man who presented with a progressively increasing swelling in the right groin, which represented a squamous cell bladder carcinoma herniating into the right inguinal canal. The manuscript discusses the role of multimodality imaging in bladder carcinoma presenting as an inguinoscrotal hernia. The patient subsequently underwent treatment with a chemotherapy regimen consisting of 5-fluorouracil and mitomycin, which was extrapolated from squamous cell carcinoma of the anal canal, and responded well.

## Introduction

Urinary bladder hernia is uncommon, comprising less than 4% cases of all inguinal hernias.^1^ The urinary bladder may herniate into the inguinal canal (most common), scrotum or femoral canal; isolated cases of urinary bladder herniation into the obturator foramen, ischiorectal fossa, as well as through the abdominal/pelvic wall have been reported. The most common risk factor is bladder outlet obstruction with chronic bladder distention and its contact with the inguinal canal. Risk is increased with advanced age, usually occurring after the fifth decade of life, probably related to decreased tone of the bladder wall; it tends to occur in obese men.^2^

Urinary bladder malignancy within the inguinal hernia is extremely rare, with a few cases reported in the literature.^3^ To the best of our knowledge, the available current literature does not mention squamous cell carcinoma (SCC) of the urinary bladder arising from the bladder hernia. In this article, we describe a rare case of an SCC of the urinary bladder presenting as an inguinoscrotal hernia.

## Case report

A 74-year-old male, non-smoker with a past medical history of hypertension, benign prostate hypertrophy (BPH) and remote post-traumatic exploratory laparotomy presented to the emergency department with progressively increasing right groin pain and swelling. These symptoms had been present for 2 years, relieved by analgesic use, but had been exacerbated over the last 2 months. The patient had accompanying macroscopic haematuria and weight loss. Examination revealed a non-reducible, large painful mass palpable in the right groin.

**FIGURE 1:**
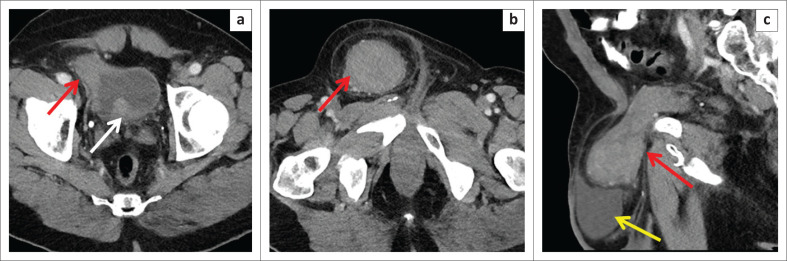
Computed tomography (CT) scan of the pelvis with intravenous contrast: (a) Axial CT image of the pelvis acquired during the portal venous phase demonstrates irregular thickening of the right anterolateral urinary bladder wall (red arrow) and an enlarged prostate bulging into the bladder base (white arrow). (b) Axial and (c) sagittal CT images of the inguinal region reveal a heterogeneous, enhancing soft tissue mass in the right inguinal canal, inseparable from the right anterolateral aspect of the urinary bladder wall (red arrow). There is a moderately sized hydrocoele in the right scrotum (yellow arrow).

Computed tomography (CT) scan of the abdomen and pelvis demonstrated a 6.5 cm × 5.4 cm × 6.4 cm enhancing soft tissue mass in the right inguinal canal, inseparable from the anterolateral aspect of the urinary bladder with an accompanying hydrocoele (Figure 1). Additionally, a few enlarged lymph nodes were noted in the pelvis (bilateral obturator and iliac groups) and retroperitoneum (left para-aortic). The largest enlarged lymph node in the pelvis was the right obturator lymph node measuring 1.2 cm × 1 cm, and the left para-aortic lymph node measuring 1.1 cm × 0.9 cm.

A cystoscopy was performed; however, the inguinal mass was not approachable. A trans-urethral biopsy of the enlarged prostate demonstrated moderately differentiated keratinising SCC with necrosis.

**FIGURE 2:**
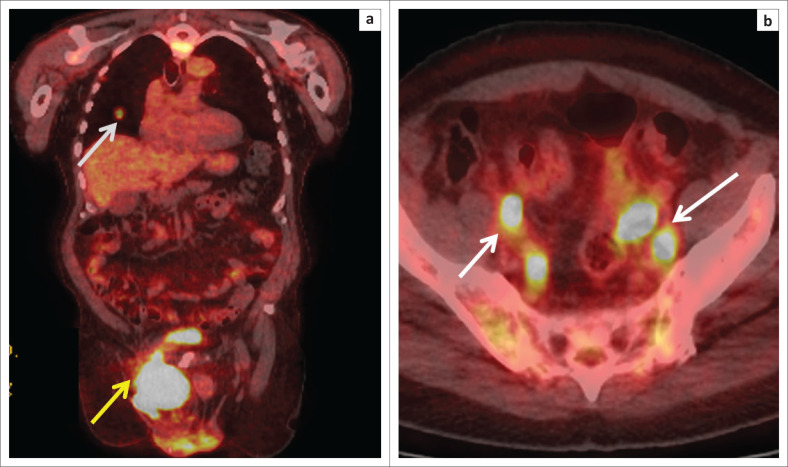
(a) Coronal 18-fluoro-D-glucose positron emission tomography/computed tomography (PET/CT) fusion image shows intense hypermetabolic activity (standardised uptake value [SUV] max 28) in the right inguinal mass (yellow arrow). A few metabolically active nodules were present in both lungs, of which one is shown in the same picture (white arrow). (b) Axial PET/CT fusion image demonstrating multiple metabolically active lymph nodes in the pelvis (white arrows) with SUV max of 10.

**FIGURE 3:**
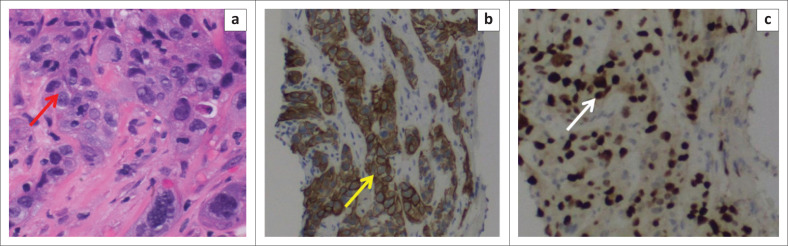
Histopathological diagnosis of primary squamous cell carcinoma of the urinary bladder (a) Hematoxylin and eosin stain; 400 × magnification indicating well differentiated squamous cells (red arrow). (b) The Ck-7 stain is positive (yellow arrow). (c) The P40 stain is also positive in the tumour cells (white arrow). The histopathology confirms the diagnosis of primary squamous cell carcinoma of the urinary bladder.

A 2-deoxy-2-[fluorine-18] fluoro-D-glucose integrated with CT (18F-FDG positron emission tomography [PET]/CT) scan demonstrated a hypermetabolic mass in the right inguinal region, with continuous extension into the urinary bladder lumen (Figure 2). However, accurate tumour differentiation was not possible because of background normal tracer excretion into the bladder. Multiple enlarged bilateral obturator, iliac and left para-aortic group of lymph nodes seen on diagnostic CT also demonstrated increased tracer activity. A few bilateral hypermetabolic pulmonary nodules were noted in keeping with metastases. Ultrasound-guided biopsy of the inguinal mass was consistent with SCC (Figure 3).

Trans-urethral resection of the prostate demonstrated SCC that was unusual, but suggested submucosal infiltration of the primary bladder carcinoma. A modified treatment protocol extrapolated from treatment of SCC of the anal canal was utilised. The patient received radiation therapy and a chemotherapy regimen consisting of 5-fluorouricil and mitomycin.

## Discussion

Most often, the urinary bladder herniates into the inguinal canal. The second most frequent location is through the femoral triangle, followed by less common sites including the obturator, perineal and umbilical regions.^4^ Three types of inguinal hernias of the urinary bladder have been described with regard to their relation to the peritoneum: (1) paraperitoneal hernia, in which the bladder remains extraperitoneal and medial to the peritoneal reflection, (2) intraperitoneal hernia, in which the bladder is completely covered by the peritoneum, and (3) extraperitoneal hernia, in which the peritoneum remains in the abdomen and the bladder alone herniates.^5^ Risk factors for inguinal bladder hernia include male gender, advanced age, chronic urinary obstruction and obesity.^2^ As with other hernias, bladder hernia occurs as a result of increased intra-abdominal pressure coupled with a loss of mechanical integrity of the muscles, tendons and other soft tissue structures.

The majority of patients with inguinal bladder hernia remain asymptomatic, thus making clinical diagnosis challenging, and dependent upon imaging findings. Symptomatic patients may present with swelling in the inguinal region, dysuria, haematuria and urinary obstruction.^2^ Although inconsistent, the classic Mery’s sign may be elicited, which is a two-step urination facilitated by applying pressure on the hernia, and disappearance of the hernia after voiding.^4^

Potential complications of bladder hernia are obstructive uropathy, incarceration, strangulation, bladder infarction secondary to incarceration and iatrogenic injury damage during hernia repair.^2^ Benign prostatic hyperplasia, hydronephrosis, vesicoureteric reflux and scrotal abscesses may be associated with bladder hernia.^2^

Squamous cell carcinoma accounts for 3% – 5% of bladder malignancies in Western countries. Our index patient demonstrated a rare case of SCC within a herniated bladder that also infiltrated the prostate gland.^6^ Predisposing factors include those that cause urothelial injury, such as indwelling catheters, chronic inflammation, calculi, smoking and urinary tract infections.^2^ Squamous cell carcinoma of the urinary bladder is also more common in countries where *Schistosoma haematobium* is endemic. In such endemic regions, patients with SCC of the bladder are 10–20 years younger at presentation than patients with transitional cell carcinoma.^7^ Additionally, SCC related to schistosomiasis is usually well differentiated, presenting as an exophytic, nodular and fungating mass with relatively low propensity for lymph nodal and distant metastases compared to SCC not related to schistosomiasis.^7^ This is believed to be caused by lymphatic and capillary fibrosis due to chronic parasite infection.

Accurate staging of bladder cancer is crucial to optimise and prognosticate the individual patient. Treatment of the bladder cancer depends on the presence or absence of muscle invasion, carcinoma *in situ* and metastases.^8^ When a trans-urethral resection of a bladder tumour (TURBT) is feasible, complete resection of the tumour is attempted, whereas radical cystectomy is the standard curative treatment for patients with muscle invasion.^8^ Patients with metastatic bladder cancer or unresectable muscle invasive tumours are treated with chemotherapy.^8^

Because of the paucity of specific symptoms, clinical diagnosis of bladder hernia is challenging. Imaging is important to diagnose a bladder hernia and its associated pathologies. It is important to diagnose the contents pre-operatively to avoid the risk of injury during surgical repair. Inguinoscrotal bladder hernias are associated with emergency complications such as obstructive uropathy and bladder infarction because of an incarcerated hernia that requires subtotal cystectomy.^9^

Computed tomography is the most common imaging modality performed for the detection and staging of bladder tumours.^10^ Computed tomography urography provides information about the anatomy, as well as physiological function, and is usually performed as a three-phase examination. Three phases of acquisition include non-contrast, nephrographic and excretory phases after intravenous administration of iodinated contrast material. The non-contrast phase can detect high-attenuation blood clots, calcifications and calculi. Acquisition of the urinary bladder in the nephrographic phase allows visualisation of an enhancing bladder tumour, which is better delineated against low-attenuation urine. At the delayed excretory phase, the tumour appears as a filling defect and the tumour may be a polypoidal, plaque-like, infiltrative or diffuse lesion.^10^ Delayed excretory phase scans are superior in delineating the inguinal bladder hernia, which is continuous with the urinary bladder lumen. Alternatively, a direct CT cystogram can be performed; however, it is invasive and requires catheterisation.

Magnetic resonance imaging provides superior contrast resolution compared to CT, making it possible to delineate the tumour from the normal detrusor muscle of the bladder wall. The detrusor muscle layer is seen as a hypointense band against hyperintense urine and perivesical fat on T2-weighted images.^11^ Muscular invasion is seen as interruption of the normal hypointense band of the detrusor muscle by an intermediate to high signal tumour on T2-weighted images. Magnetic resonance imaging has a 91% sensitivity and 96% specificity in differentiating ≤ T1 tumours (non-muscle invasive) from ≥ T2 tumours (muscle invasive) prior to surgery.^10,11^ Newer functional MRI techniques, such as diffusion-weighted imaging (DWI) and dynamic contrast-enhanced (DCE) imaging, increase the accuracy of local staging.

Both CT and MRI have low sensitivity in detecting abdominal or pelvic lymph node metastasis. Pelvic nodes greater than 8 mm and abdominal nodes greater than 10 mm should be regarded as pathologically enlarged.^10,11^ Staging of pulmonary, liver and lymph nodal metastasis is usually performed with CT. Detection of the primary bladder tumour on 18F-FDG PET/CT is often difficult because of the intense accumulation of excreted FDG tracer in the urine.^12^ Similar problems exist when evaluating tumour developing within a bladder diverticulum or bladder hernia. A careful inspection of the non-diagnostic CT images may detect extravesical extension of the tumour, which indicates T3b disease.^12^ 2-deoxy-2-[fluorine-18] fluoro-D-glucose integrated with CT is most useful in the evaluation of lymph node or distant metastasis, and recurrence. Increased FDG tracer activity within the adjacent organs, such as the prostate, vagina, cervix, uterus and rectum, may help in establishing T4a disease. Computed tomography and MRI are also useful tools in monitoring the effects of treatment.^12,13^

In our index patient, a biopsy during cystoscopy was not amenable because of the size and position of the hernia. Instead, a trans-urethral resection of the prostate with a biopsy was performed, revealing metastases from the SCC of the bladder.

## Conclusion

To the best of our knowledge, SCC of the urinary bladder hernia has not been previously reported. Imaging plays a vital role in diagnosis and management of such unusual presentations. The role of multimodality imaging techniques, as well as imaging features, has been described in this article.
